# Transcripts and tumors: regulatory and metabolic programming during biotrophic phytopathogenesis

**DOI:** 10.12688/f1000research.16404.1

**Published:** 2018-11-19

**Authors:** Lara Schmitz, Sean McCotter, Matthias Kretschmer, James W. Kronstad, Kai Heimel

**Affiliations:** 1 Institute for Microbiology and Genetics, Department of Molecular Microbiology and Genetics, Georg-August-University-Göttingen, Göttingen, Lower Saxony, D-37077, Germany; 2 Michael Smith Laboratories, Department of Microbiology and Immunology, University of British Columbia, Vancouver, BC, V6T 1Z4, Canada

**Keywords:** Ustilago maydis, transcriptome, RNAseq, pathogenicity, regulators, effectors, nutrients, metabolism

## Abstract

Biotrophic fungal pathogens of plants must sense and adapt to the host environment to complete their life cycles. Recent transcriptome studies of the infection of maize by the biotrophic pathogen *Ustilago maydis* are providing molecular insights into an ordered program of changes in gene expression and the deployment of effectors as well as key features of nutrient acquisition. In particular, the transcriptome data provide a deeper appreciation of the complexity of the transcription factor network that controls the biotrophic program of invasion, proliferation, and sporulation. Additionally, transcriptome analysis during tumor formation, a key late stage in the life cycle, revealed features of the remodeling of host and pathogen metabolism that may support the formation of tremendous numbers of spores. Transcriptome studies are also appearing for other smut species during interactions with their hosts, thereby providing opportunities for comparative approaches to understand biotrophic adaptation.

## Introduction

**Figure 1.**
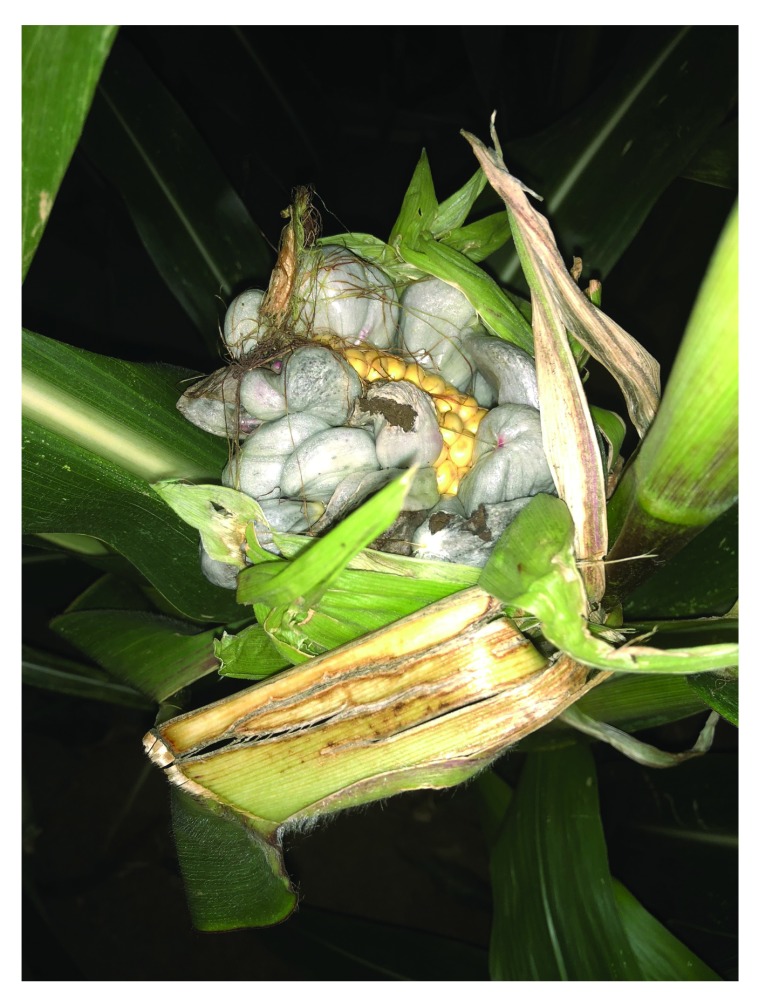
Tumor formation on maize by *Ustilago maydis*. An infected maize cob found in a cornfield close to Göttingen, Germany, in September 2018. At the end of the growing season, infected kernels give rise to greatly enlarged and bulbous plant tumors filled with black teliospores. Tumors can develop on all aerial parts of the plant but are most prominent in infected cobs.

Fungal phytopathogens in the order Ustilaginales generally attack cereal and grass plants to cause smut diseases, so named because of the tremendous masses of sooty spores produced in infected tissue ( Figure 1). Biotrophic pathogens such as the smut fungi are dependent on living hosts to complete their life cycles. Effective adaptation to the host environment is therefore critical for overcoming the plant immune response and successfully exploiting host nutrients through remodeling of metabolism and effective competition. The mechanisms by which biotrophic pathogens manipulate host metabolism to divert carbon, nitrogen, or micronutrients such as iron for their own use are starting to be identified ^1– 4^. Among the Ustilaginales, the *Zea mays* (maize) pathogen *Ustilago maydis* has emerged as an experimentally tractable model for studying the adaptation of biotrophic fungal pathogens to the host environment. As an example of the intricate interaction with the plant host, the life cycle of *U. maydis* involves germination of diploid teliospores on host tissue with subsequent mating between haploid meiotic progeny to form an invasive filamentous cell type ^5, 6^. The filaments then form appressoria (invasion structures) to penetrate host tissue with subsequent extensive proliferation, induction of tumors (galls), and eventual formation of melanized teliospores ^5, 7^ ( Figure 1). A large number of effectors are predicted for *U. maydis*, and these proteins are thought to play key roles in managing the infection process. To date, characterized effectors include Cmu1, Pit2, Pep1, and Tin2, which influence host defense, and See1 and Rsp3, which influence tumor progression and the fungal response to host defense, respectively ^3, 8^.

In this review, we focus on recent genome-wide transcriptome studies of the infection process that have provided insights into the transcriptional regulation associated with disease, the deployment of effectors by *U. maydis* to manage the infection process, and the remodeling of host metabolism during fungal proliferation. Although not covered here, earlier studies also examined the transcriptome of *U. maydis* in culture and during infection ^6, 7, 9– 23^. We also refer readers to a wealth of primary literature and recent reviews on the role of transcription factors, regulators, and effectors in the disease process for *U. maydis* ^13, 14, 20, 24– 27^.

## Transcriptome analysis throughout the infection process

**Figure 2.**
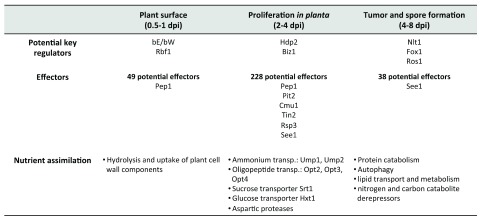
Regulators, effectors, and metabolism during *Ustilago maydis* infection of maize. Overview of transcriptional regulation, the timing of effector function, and metabolic changes and requirements during early, middle, and late stages of the *U. maydis*–maize interaction. DPI, days post inoculation.

A recent time-resolved and genome-wide study of transcriptional changes occurring during the *U. maydis*–maize interaction provides detailed insights into the processes underlying infection, tumor formation, and sporogenesis ^28^ *.* Among 14 regulatory modules identified, three gene sets/developmental programs were specifically associated with virulence and were upregulated during discrete developmental stages ( Figure 2). These gene sets are expressed during 1) growth on the plant surface (early), 2) biotrophic development *in planta* (middle), and 3) tumor formation (late/sporogenesis). In all three sets, genes encoding secreted proteins (effectors) are significantly overrepresented, emphasizing their critical role during biotrophic development. Metabolic changes are also predicted and, importantly, several transcription factors with a strong connection to each respective module were identified as potential key regulators ( Figure 2).

For the early stage of infection, it is known that gene regulation by the zinc finger transcription factor Rbf1 is required for the initial steps of pathogenic development, including filament formation, induction of G2 cell cycle arrest, and appressoria-mediated plant penetration ^6, 25^. Recent work by Lanver *et al*. ^28^ revealed that Rbf1 shows a strong regulatory connectivity to genes expressed at the early stage (0.5–1 days post inoculation [DPI]). Among the 398 genes that are upregulated, 49 encode secreted proteins that are enriched for hydrolytic enzymes with a predicted function in appressoria-mediated plant penetration. Several genes induced on the leaf surface encode sugar- or nitrogen-related transporters, indicating priming of *U. maydis* for altered nutrient availability during plant colonization.

The subsequent middle phase of biotrophic development (2–4 DPI) involves the re-initiation of fungal proliferation, establishment of a compatible biotrophic interaction, and the initiation of tumor formation. Enriched functional categories in the transcriptome include nitrogen and carbon source-related processes and components of the secretory pathway including the unfolded protein response (UPR) ^28^. Transcriptional regulation during the biotrophic stage is modulated by direct interaction between UPR components and key regulators of fungal development and pathogenicity ^27^. Potential transcriptional regulators of this stage include the Rbf1-regulated homeodomain transcription factor Hdp2 ^6, 7^ and the C2H2-zinc finger transcription factor Biz1 ^24^.

Among the 228 genes encoding putative effectors that were upregulated in the middle stage, 153 lack functional signatures. However, the characterized genes encoding the effectors Pep1, Pit2, Cmu1, See1, Tin2, and Rsp3 are highly induced at this stage, and these are required for full virulence of *U. maydis* and/or the suppression of plant defense reactions and fungal defense, respectively (reviewed in 3). Organ- and cell type-specific transcriptome analysis revealed that genes encoding the core effectors Pep1, Pit2, Cmu1, and Tin2 are expressed independent of the plant organ or cell type in leaves and tassels, whereas See1 is exclusively expressed in leaf tissue ^29, 30^. Tumor formation occurs by distinct mechanisms in hyperplastic bundle sheath-derived tumor cells (HPT) or hypertrophic mesophyll-derived tumor cells (HTT) ^31^. See1 mediates only bundle sheath cell-derived tumor formation, whereas mesophyll-derived tumor formation is independent of See1 ^31^.

During tumor formation in the middle stage, the ammonium transporters Ump1 and Ump2 and the oligopeptide transporters Opt2, Opt3, and Opt4 appear to mediate nitrogen acquisition *.* Ump1, Ump2, and Opt2 are induced already on the leaf surface and further upregulated *in planta* ^28, 32^. The fact that two secreted aspartic proteases are co-expressed with the OPTs suggests that the breakdown of extracellular proteins and peptide uptake may be part of the biotrophic developmental program of *U. maydis* ^28^. Carbon assimilation during biotrophic development is known to occur mainly via the high-affinity sucrose and glucose transporters Srt1 and Hxt1, respectively. While Srt1 is strongly induced during plant colonization, Hxt1 expression is independent of the fungus–plant interaction ^33, 34^.

At later stages of disease (4–8 DPI), a third wave of effectors supports tumor maturation and the production of melanized teliospores. One important regulator after establishment of the biotrophic interaction is the APSES transcription factor Nlt1. Nlt1 appears to guide tumor formation on leaf tissue but not anthocyanin and tumor formation on the base of the stem ^28^. Another factor contributing to the regulation of effector gene expression at the later stage is the forkhead transcription factor Fox1. Fox1 is required for the attenuation of plant defense responses and for full expression of 141 genes, of which 38 encode potential effectors, as well as several proteins involved in sugar processing and transport and secondary metabolism ^14^. Genes encoding effectors that are specifically expressed at late stages of biotrophic development are largely unexplored functionally. Potentially, they function to sustain tumor development and guide spore formation as well as the formation of a mucilaginous matrix in which spores are embedded. A key regulator of these late-stage events is the DNA-binding WOPR protein Ros1. Ros1 function is essential for teliosporogenesis, and the majority of Ros1 target genes are involved in metabolism and cellular transport. The Ros1-dependent induction of late effector genes and repression of early effector-encoding genes indicates that Ros1-mediated regulation occurs by direct and indirect mechanisms, involving additional regulators ^20^. As described below, further insights into late-stage changes in both host and pathogen gene regulation were obtained by examining transcriptomes specifically in tumors.

## Transcriptome changes reveal extensive metabolic remodeling in tumors

Recent transcriptome profiling experiments of the late stage of infection, when tumors were evident, identified host genes that were upregulated (4,086) or downregulated (5,237) ^4, 35^. Downregulated functional categories included metabolic pathways for amino acids, organic acids, lipids, and photosynthesis. By contrast, upregulated functional categories were related to plant defense and lipid and carbohydrate metabolism. As expected for proliferating maize cells during tumor formation, meristem maintenance functions were upregulated during infection. Additionally, maize transcription factors that are normally expressed in leaf tissue during plant development were downregulated in infected plants. In contrast, transcription factors normally expressed in flowers were upregulated in tumor tissue. Thus, *U. maydis* appears to inhibit the transition to adult tissue development and instead induces a flowering program. Chloroplast functions related to vegetative tissue, such as photosynthesis, lipid production, amino acid formation, and secondary metabolism, were downregulated while reproductive functions such as sucrose and starch metabolism were upregulated during infection. Maize mutants in host components related to the vegetative to flowering transition, the biogenesis of the chloroplast, or starch formation all showed altered susceptibility to *U. maydis*. For example, impaired chloroplast formation in the why1 mutant led to increased susceptibility, while the inactive/delayed transition to a flowering state (id1) and the reduced starch formation leading to lower sink capacity (su1) led to increased plant resistance ^4, 35^.

Consistent with earlier work, transcriptome profiling of tumor tissue supported an important role of sugar transporters for metabolic exploitation of the host ^4, 13, 33^. Hexoses and other sugars represent major carbon sources for *U. maydis*, and the fungus possesses 19 proteins with similarities to sugar/hexose-like transporters. As mentioned above, transporters such as Hxt1 and Srt1 play important roles during infection. Hxt1 is constitutively expressed and has transport activity mainly for glucose but also fructose and mannose. As a high-affinity glucose transporter, Hxt1 also possesses glucose-sensing activity and competes with the host for glucose molecules freed from sucrose by invertase. Previous work involving the deletion of the gene for this transporter or expression of a constitutive active (sensing but not transport activity) version resulted in strains with reduced or fully abolished virulence, respectively ^33^. As indicated, the transporter Srt1 is induced only *in planta* and transports sucrose. Based on its extremely high affinity to sucrose (up to 200-fold versus a typical plant transporter), it outcompetes the sucrose transport functions of the host. The virulence of deletion strains for *srt1* was strongly reduced ^33^. Deletion of both *hxt1* and *srt1* further reduced fungal virulence, thus indicating a simultaneous uptake and use of sucrose and glucose by the pathogen during infection. Other carbon sources such as organic acids (fatty acids or carboxylic acids) may also contribute to the carbon supply of the pathogen at early time points of infection on the cuticle and at a late stage during spore formation ^36^. Monocarboxylic acids are a carbon source for *U. maydis in vitro* but can have detrimental effects on fungal longevity and reactive oxygen species resistance ^37^. *Ustilago* is known to inhibit the transition to C4 metabolism that is dependent on the dicarboxylate malate for transport between cells. However, it is still unknown how and if different host-derived carboxylic acids affect fungal virulence ^2^.

In general, the transcriptome analyses of infected tissue support the idea that control of chloroplast functions is a critical aspect of the infection of maize by *U. maydis*. Moreover, primary and secondary metabolic functions and plant defense contributions of the chloroplast appear to be suppressed or altered during infection. In line with these observations, chloroplasts were observed to accumulate high amounts of starch, consistent with an overall increased starch content in tumor tissue ^1, 4, 31^. However, the mechanisms by which *U. maydis* impacts the chloroplast are still unknown.

## Beyond *Ustilago maydis*: transcriptome studies with other smut fungi

A number of transcriptome studies have also been carried out for host–pathogen interactions involving other species in the Ustilaginales. For example, Que *et al*. ^38^ examined the interaction between the sugarcane smut *Sporisorium scitamineum* and its host *Saccharum officinarum*, comparing the transcriptional responses of resistant (Yacheng05-179) and susceptible (“ROC”22) plant lines. This study focused on the transcriptional responses of the host at 24, 48, and 120 hours after inoculation (HAI) in the resistant and susceptible cultivars. Both lines showed an overall increase in the number of differentially expressed genes (DEGs) as infection progressed, but the resistant cultivar exhibited an earlier response to pathogen infection. Consistently, most DEGs were associated with stress and defense responses to pathogen attack. The resistant cultivar revealed a rapid peak in DEGs at 48 hours, and more DEGs were associated with disease resistance when compared to the susceptible cultivar. The susceptible cultivar showed a similar accumulation of disease resistance-associated DEGs at the 120-hour time point, highlighting the difference in the timing of the response to infection by the resistant and susceptible lines. Categories of genes showing differential expression included metabolic functions associated with resistance, hormone signaling, flavonoid biosynthesis, cell wall fortification, and defense-related transcription. Interestingly, a total of 26 host chitinases were differentially expressed in the *S. scitamineum-*infected plants. Expression of two of the chitinases in tobacco led to increased disease resistance against *Fusarium solani* and *Botrytis cinerea*.

In a more recent transcriptome study, Wang *et al*. ^39^ investigated the interaction between the edible culm gall-forming fungus *Ustilago esculenta* and the host *Zizania latifolia*, an aquatic grass. The transcriptome study compared infections for two strains of the fungus: the teliospore-forming strain Huijiao and the non-sporulating strain Jiaobai. In general, DEGs were more frequently observed in plants infected with the Jiaobai strain versus the Huijiao strain, although the proportion of fungal transcripts was lower in the former infection. Huijiao-infected plants exhibited a peak of 57% of total transcripts of fungal origin at 10 days after swelling (DAS) compared to only ~5% for Jiaobai.

With regard to host gene expression, clear differences were observed over the course of infection with sampling every 4 days starting at 7 days prior to swelling and ending at 13 DAS. That is, for plants infected with the Jiaobai and Huijiao strains, host genes were identified that were upregulated at all three stages as well as host genes that were upregulated only at specific time points in comparison to uninfected controls. Additionally, 170 and 205 host DEGs with potential roles in culm gall formation were identified at 1 and 10 DAS, respectively. These genes encoded proteins with predicted roles in primary and secondary metabolism as well as in plant–pathogen interactions and plant hormone signal transduction.

An analysis of pathogen genes that were upregulated was also carried out to attempt to identify functions involved in culm gall formation. Applying an analysis that paralleled that used for host genes, 78 and 109 pathogen genes were identified at 1 and 10 DAS, respectively. A significant fraction of these genes lacked annotation, while genes coding for proteins had predicted functions in nucleotide binding, transcriptional regulation, hydrolase activity, transferase activity, and transport.

In addition to *U. maydis*, other members of the Ustilaginales have been investigated as models for studying biotrophic phytopathology. For example, Rabe *et al*. ^40^ carried out a comprehensive study of the genome, transcriptome, and usefulness as a model system of the grass pathogen *Ustilago bromivora* and one of its hosts, *Brachypodium* spp. Initially, a susceptible line, Bd28, was identified after screening a number of *Brachypodium* spp. accessions for susceptibility to *U. bromivora*. In addition to extensive characterization of *U. bromivora* and its interaction with the host, a transcriptome analysis compared fungal gene expression of *U. bromivora* in axenic culture with infected stems of Bd28. It was found that 7.3% of the identified fungal transcripts were upregulated while 16.8% were downregulated. It is notable that these percentages are far lower than those reported by Lanver *et al*. ^28^ for differential expression of *U. maydis* genes during plant infection. For *U. bromivora*, transcripts encoding potential effectors were enriched among upregulated transcripts during infection (30.8%). In comparison, only 5.7% of transcripts (genes) overall were predicted to encode secreted proteins. Among the genes for predicted secreted proteins, 84.1% encoded proteins of unknown function compared with a frequency of 46.4% for all of the proteins encoded by *U. bromivora*. This finding is consistent with other studies indicating that most secreted effectors of plant pathogenic fungi are uncharacterized and poorly conserved between species ^28^. The observation that all of the functionally characterized effectors identified in *U. maydis* also have homologs in *U. bromivora* highlights the importance of fungal model systems to investigate molecular principles of effector function.

## Conclusions and future perspectives

Overall, recent transcriptome profiling has revealed dynamic changes in the transcription of pathogen genes encoding metabolic functions (e.g. for nitrogen and carbon metabolism) that likely reflect shifting environmental conditions throughout the disease process as *U. maydis* colonizes tissue, induces tumors, and sporulates. It is fascinating that the changes in pathogen gene expression are integrated with and driven by effector-mediated manipulation of host cell metabolism and plant immunity. Time-resolved analyses greatly increase our understanding of individual stages of pathogenic development and how transitions between those are coordinated. Future work will exploit the accumulated wealth of data to experimentally address predictions about pathogen effectors and their regulators and the importance of specific metabolic changes for both the pathogen and the host. For example, it is of keen interest to understand how different host environments are sensed and translated into adapted transcriptional programs. Also, future work will further examine the details of tumor formation. It is important to mention that these studies will benefit from technical improvements that increase the detection of less-expressed genes and reproducibility in cell type-specific transcriptome studies. Moreover, comparisons of non-tumor-forming smuts, such as *Sporisorium reilianum* and *Ustilago hordei,* with *U. maydis* may provide clues about the timing of tumor induction and the corresponding specific pathogen and host functions. The sophisticated molecular tools established for *U. maydis,* and the emerging tools for *U. bromivora*, will provide novel insights that are relevant specifically for tumor induction and sporulation by other smut fungi as well as generally for the mechanisms of biotrophy.

## Abbreviations

DAS, days after swelling; DEGs, differentially expressed genes; DPI, days post inoculation; UPR, unfolded protein response
